# The effect of antifibrotic agents on acute respiratory failure in COVID-19 patients: a retrospective cohort study from TriNetX US collaborative networks

**DOI:** 10.1186/s12890-024-02947-5

**Published:** 2024-04-02

**Authors:** Hsin-Yi Wang, Shih-Chuan Tsai, Yi-Ching Lin, Jing-Uei Hou, Chih-Hao Chao

**Affiliations:** 1 Department of Nuclear Medicine, Taichung Veterans General Hospital, Taichung, Taiwan; 2 School of Medicine, National Defense Medical Center, Taipei, Taiwan; 3 Department of Medical Imaging and Radiological Technology, Institute of Radiological Science, Central Taiwan University of Science and Technology, Taichung, Taiwan; 4 Department of Public Health, China Medical University, Taichung, Taiwan; 5 Division of Chest Medicine, Department of Internal Medicine, Chang Bing Show Chwan Memorial Hospital, Changhua, Taiwan

**Keywords:** Antifibrotic agent, Nintedanib, Pirfenidone, COVID-19, Acute respiratory failure, Mortality

## Abstract

**Background:**

The coronavirus disease 2019 (COVID-19) pandemic has had a significant impact on global health and economies, resulting in millions of infections and deaths. This retrospective cohort study aimed to investigate the effect of antifibrotic agents (nintedanib and pirfenidone) on 1-year mortality in COVID-19 patients with acute respiratory failure.

**Methods:**

Data from 61 healthcare organizations in the TriNetX database were analyzed. Adult patients with COVID-19 and acute respiratory failure were included. Patients with a pre-existing diagnosis of idiopathic pulmonary fibrosis before their COVID-19 diagnosis were excluded. The study population was divided into an antifibrotic group and a control group. Propensity score matching was used to compare outcomes, and hazard ratios (HR) for 1-year mortality were calculated.

**Results:**

The antifibrotic group exhibited a significantly lower 1-year mortality rate compared to the control group. The survival probability at the end of the study was 84.42% in the antifibrotic group and 69.87% in the control group. The Log-Rank test yielded a p-value of less than 0.001. The hazard ratio was 0.434 (95% CI: 0.264–0.712), indicating a significant reduction in 1-year mortality in the antifibrotic group. Subgroup analysis demonstrated significantly improved 1-year survival in patients receiving nintedanib treatment and during periods when the Wuhan strain was predominant.

**Discussion:**

This study is the first to demonstrate a survival benefit of antifibrotic agents in COVID-19 patients with acute respiratory failure. Further research and clinical trials are needed to confirm the efficacy of these antifibrotic agents in the context of COVID-19 and acute respiratory failure.

**Supplementary Information:**

The online version contains supplementary material available at 10.1186/s12890-024-02947-5.

## Background

Coronavirus disease 2019 (COVID-19), caused by the novel coronavirus SARS-CoV-2, has had a major impact on global health and economies [1]. As of May 2023, there have been more than 760 million confirmed infections and 6.9 million deaths worldwide [2].

SARS-CoV-2 mainly infects the respiratory system and causes mild symptomatic or asymptomatic disease. However, approximately 14% of patients will require oxygen and hospital treatment, and approximately 5–6% will progress to severe pneumonia or acute respiratory distress syndrome (ARDS) and require intensive care unit (ICU) treatment [3]. The mortality rate in COVID-19 patients with ARDS requiring mechanical ventilation ranges from 35.7 to 94% [4, 5].

An emerging complication of COVID-19 is pulmonary fibrosis [6, 7]. Several studies have suggested that pulmonary fibrosis is a common complication that results in poor survival and functional outcomes in COVID-19 patients [8, 9]. Novel antifibrotic agents such as nintedanib and pirfenidone have demonstrated effectiveness in managing patients with idiopathic pulmonary fibrosis (IPF). Nintedanib functions as an intracellular inhibitor targeting vascular endothelial growth factor receptors 1–3, fibroblast growth factor receptors 1–3, and platelet-derived growth factor receptors a and b [10]. On the other hand, pirfenidone is an orally bioavailable pyridone derivative known for its anti-inflammatory, antifibrotic, and antioxidant properties [11]. Both medications have been proven to reduce the rate of annual decline in forced vital capacity and mortality in patients with IPF [12–15]. Nintedanib also exhibits inhibitory effects on fibrogenesis across various pulmonary disorders, including connective tissue-associated interstitial lung diseases [16]. Notably, a recent study showed that antifibrotic therapy had similar efficacy in treating patients with progressive pulmonary fibrosis regardless of whether the underlying disease was IPF or non-IPF [17]. Novel antifibrotic agents are potential treatments for COVID-19-induced acute respiratory failure. We conducted this retrospective study to investigate the effect of antifibrotic agents on patients with acute respiratory failure due to COVID-19.

## Methods

### Setting

In this retrospective cohort study, we utilized the US Collaborative Network in the TriNetX database, which comprises 61 healthcare organizations (HCOs). TriNetX is a global federated health research network that provides access to electronic medical records, including diagnoses, procedures, medications, laboratory values, and genomic information across large HCOs.

The TriNetX platform complies with the Health Insurance Portability & Accountability Act and the General Data Protection Regulation. The Western Institutional Review Board has granted TriNetX a waiver of informed consent, as the platform only aggregates counts and statistical summaries of deidentified information.

### Cohort

**Fig. 1:**
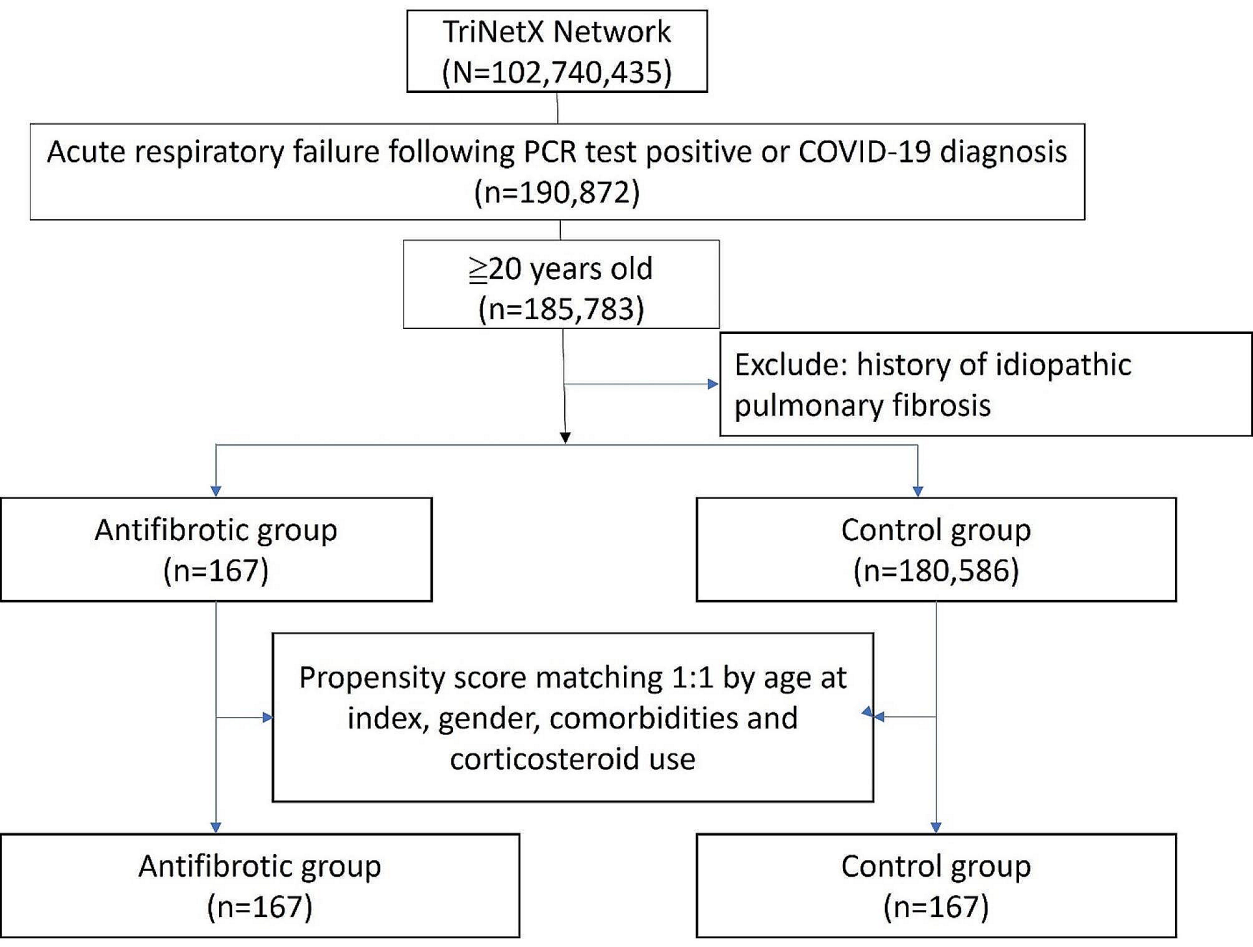
A flowchart of the cohort construction from participants

We included adult patients (≥ 20 years old) with a positive SARS-CoV-2 PCR test or a COVID-19 diagnosis (ICD10: U07.1, U07.2, or J12.82) during the study period in the TriNetX database. Patients with a past medical history of idiopathic pulmonary fibrosis (ICD10: J84.12) prior to their COVID-19 diagnosis were excluded. The index date was set as the day acute respiratory failure (ICD10: J96.00, J96.0, J96.01, and J96.02) developed within 3 days before to 1 month after the COVID-19 diagnosis. The study population was then divided into 2 groups: those who received oral antifibrotic treatment with nintedanib or pirfenidone (antifibrotic group) and those who did not receive antifibrotic treatment (control group). A flowchart of the cohort construction from participants enrolled between 1 June 2019 and 23 August 2023 is shown in Fig. 1. The main outcome of this study was 1-year mortality. Detailed query criteria are shown in Additional file 1.

Subgroup analysis was conducted to compare 1-year mortality rates between patients treated with nintedanib and those who received no antifibrotic agent, as well as between patients treated with pirfenidone and those who received no antifibrotic agent. Detailed query criteria are shown in Additional file 2 and 3. Additionally, we examined 1-year mortality rates between the antifibrotic and control groups using the same method but within different time frames to assess the effects of antifibrotic treatment across various COVID-19 strains. The study period was divided into four distinct time frames based on dominant strains in America: Whhan strain (December 2019 to April 2021, 88 patients), Alpha strain (May 2021 to June 2021, 29 patients), Delta strain (July 2021 to October 2021, 48 patients), and Omicron strain (November 2021 to August 2023, 95 patients). Detailed query criteria are shown in Additional file 4–7.

### Statistical analyses

We performed propensity score matching at a 1:1 ratio on age at index, gender, comorbidities, and corticosteroid use. We used the TriNetX built-in function to compare outcomes. We calculated the hazard ratio (HR) of 1-year mortality for both groups. We tested the proportional hazard assumption using the generalized Schoenfeld approach built in the TriNetX platform. We used the Kaplan‒Meier method for the survival probability. We defined statistical significance as a p value < 0.05. A 95% confidence interval (95% CI) was also considered evidence of statistical significance.

## Results

### Baseline characteristics of the study population

Table 1 summarizes the demographic and lifestyle characteristics, comorbidities, adrenal corticosteroid, antiviral agents and biologic agents use in the antifibrotic and control groups before and after propensity score matching. The mean age of the population in both groups was approximately 64.5 years in the antifibrotic group and 65.8 years in the control group at the index date after matching. Approximately 59% of the individuals were male, and Caucasian was the predominant race (75.4% and 64.1% in the antifibrotic and control groups, respectively). The two groups were well matched regarding demographic, lifestyle, comorbidity characteristics. The propotion of patients receiving corticosteroid and remdesivir were similar in both groups. However, a significantly higher percentage of individuals in the antifibrotic group used Tocilizumab (6% vs. 0%, respectively, *p* = 0.001) (Table 1).

**Table 1:** Antifibrotic group (Cohort 1, *N* = 167) and Control group (cohort 2, *N* = 180,586) characteristics before and after propensity score matching

Before propensity score matching							After propensity score matching				
Cohort			Patients	Mean ± SD	P-Value	Std diff.	Mean ± SD	Patients	P-Value	Std diff.	
1	AI	Age at Index	167	64.5 +/- 11.6	0.126	0.137	64.5 +/- 11.6	167	100%	0.323	
2			180,586	62.6 +/- 16.5			65.8 +/- 11.8	167	100%		
Cohort			Patients	% of Cohort	P-Value	Std diff.	Patients	% of Cohort	P-Value	Std diff.	
1	2106-3	White	126	75.4%	<0.001	0.288	126	75.4%	0.024	0.250	
2			117,798	62.2%			107	64.1%			
1	2054-5	Black or African	18	10.8%	0.132	0.124	18	10.8%	0.011	0.281	
2		American	28,266	14.9%			35	21.0%			
1	M	Male	99	59.3%	0.073	0.140	99	59.3%	1	<0.001	
2			99,085	52.3%			99	59.3%			
1	F	Female	64	38.3%	0.198	0.101	64	38.3%	1	<0.001	
2			81,886	43.3%			64	38.3%			
1	2028-9	Asian	10	6.0%	0.100	0.111	10	6.0%	1	<0.001	
2			6,835	3.6%			10	6.0%			
1	2131-1	Other Race	10	6.0%	0.129	0.104	10	6.0%	1	<0.001	
2			7,106	3.8%			10	6.0%			
Cohort			Patients	% of Cohort	P-Value	Std diff.	Patients	% of Cohort	P-Value	Std diff.	
1	I20-I25	Ischemic heart	49	29.3%	0.003	0.215	49	29.3%	1	<0.001	
2		diseases	38,072	20.1%			49	29.3%			
1	E08-E13	Diabetes mellitus	46	27.5%	0.849	0.015	46	27.5%	1	<0.001	
2			50,899	26.9%			46	27.5%			
1	E40-E46	Malnutrition	11	6.6%	0.141	0.102	11	6.6%	0.822	0.025	
2			8,104	4.3%			10	6.0%			
1	C34	Malignant neoplasm of bronchus and lung	10	6.0%	<0.001	0.259	10	6.0%	1	<0.001	
2			2,272	1.2%			10	6.0%			
1	Z87.891	Personal history of nicotine dependence	39	23.4%	0.003	0.210	39	23.4%	0.282	0.118	
2			28,627	15.1%			31	18.6%			
1	Z72.0	Tobacco use	10	6.0%	0.059	0.125	10	6.0%	1	<0.001	
2			6,347	3.4%			10	6.0%			
1	K70	Alcoholic liver	10	6.0%	<0.001	0.286	10	6.0%	1	<0.001	
2		disease	1,597	0.8%			10	6.0%			
Cohort			Patients	% of Cohort	P-Value	Std diff.	Patients	% of Cohort	P-Value	Std diff.	
1	HS050	ADRENAL	114	68.3%	<0.001	0.393	114	68.3%	1	<0.001	
2		CORTICOSTEROIDS	93,286	49.3%			114	68.3%			
1	3264	dexamethasone	61	36.5%	0.015	0.181	61	36.5%	0.219	0.135	
2			53,188	28.1%			72	43.1%			
1	6902	methylprednisolone	61	36.5%	<0.001	0.288	61	36.5%	0.246	0.127	
2			44,376	23.4%			51	30.5%			
1	5492	hydrocortisone	17	10.2%	0.680	0.031	17	10.2%	0.312	0.111	
2			17,517	9.3%			23	13.8%			
1	8640	prednisone	62	37.1%	<0.001	0.382	62	37.1%	0.164	0.153	
2			38,179	20.2%			50	29.9%			
1	2,284,718	remdesivir	10	6.0%	0.592	0.040	10	6.0%	1	<0.001	
2			9,611	5.1%			10	6.0%			
1	612,865	tocilizumab	10	6.0%	<0.001	0.322	10	6.0%	0.001	0.357	
2			760	0.4%			0	0%			
1	2,047,232	baricitinib	10	6.0%	<0.001	0.318	10	6.0%	1	<0.001	
2			843	0.4%			10	6.0%			

### Mortality

**Fig. 2:**
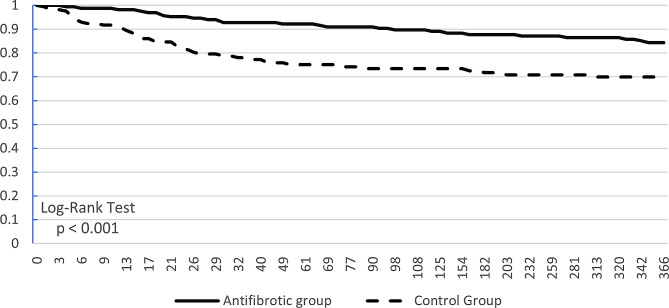
Kaplan – Meier survival curve of 1-year mortality

Figure 2 shows the Kaplan‒Meier curve of survival probability. The survival probability at the end of the study was 84.42% in the antifibrotic group and 69.87% in the control group. The Log-Rank test yielded a p value of less than0.001. The hazard ratio was 0.434 (95% CI: 0.264–0.712). The chi-square was 6.721, and the p value was 0.01 for the proportionality assessment (Table 2). The raw data for the Kaplan-Meier graph was shown in additional file 8.

**Table 2:** Kaplan – Meier survival analysis of 1-year mortality

	Patients in cohort	Patients with outcome	Median survival (days)	Survival probability at end of time window		
Antifibrotic Group	166	25	--	84.42%		
Control Group	163	42	--	69.87%		
	χ^2^	df	p			
Log-Rank Test	11.556	1	< 0.001			
	Hazard Ratio	95% CI	χ^2^	df	p	
Hazard Ratio and Proportionality	0.434	(0.264, 0.712)	6.721	1	0.010	

**Fig. 3:**
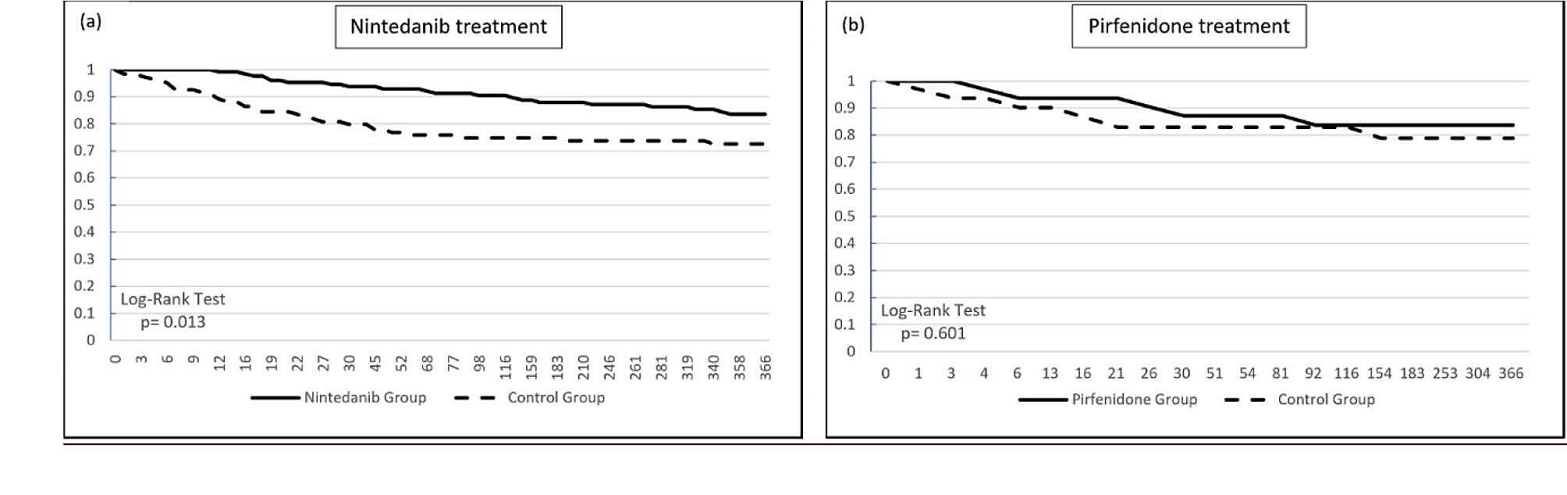
Kaplan – Meier survival curve of 1-year mortality in (a) nintedanib and (b) pirfenidone treatment group

Subgroup analysis revealed that out of the total participants, 127 patients received nintedanib, while 39 patients received pirfenidone. We observed a statistically significant improvement in 1-year mortality rates in the nintedanib group compared to the control group (*p* = 0.013). Although there was a noted improvement in the pirfenidone group, it was not statistically significant (*p* = 0.601) (Fig. 3a and b). The raw data for the Kaplan-Meier graph was shown in additional file 9 and 10.

**Fig. 4:**
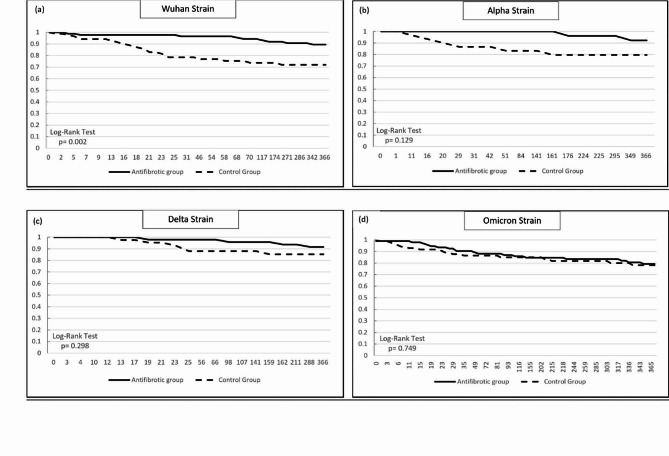
Kaplan – Meier survival curve of 1-year mortality in (a) Wuhan strain, (b) Alpha strain, (c) Delta strain, and (d) Omicron strain time frame

After dividing the study period into four distinct time frames, our analysis indicated better survival rates in the antifibrotic group across all time frames, with statistical significance observed only in the Wuhan strain period (*p* = 0.002) (Fig. 4a-d). The raw data for the Kaplan-Meier graph was shown in additional file 11–14.

## Discussion

The present retrospective multi-institutional study was conducted by the U.S. The Collaborative Research Network has demonstrated that the novel antifibrotic agents nintedanib and pirfenidone effectively reduce the 1-year mortality rate among COVID-19 patients suffering from acute respiratory failure. Remarkably, this study represents, to our knowledge, the first instance of survival benefit observed.

We observed significantly better 1-year survival in patient receiving nintedanib compared to those receiving pirfenidone. However, only 39 patients received pirfenidone in our cohort, indicating that small sample size may have contributed to insignificant results. Likewise, the small sample size during Alpha strain and Delta strain periods could also have led to insignificant findings. The characteristics of the Omicron strain, including its tendency to infect the upper respiratory tract rather than the lower respiratory tract and its lower IL-6 secretion, may have influenced the effectiveness of antifibrotic agents [18]. However, due to limitations of the TriNetX database, which does not specify the virus strain patients were infected with, comparing the effects of antifibrotic treatment across different strains was challenging. There likely were cases of mixed-strain infection across the four time frames. As a result, we are unable to ascertain the actual effect of antifibrotic agents on different virus strain. Further studies are warranted to validate these findings.

Our result revealed a higher percentage of patients in the antifibrotic group received Tocilizumab. Elevated IL-6 concentrations are associated with severe outcomes in COVID-19 [19]. Tocilizumab, an IL-6 receptor antagonist, has been demonstrated to reduce inflammatory responses and improve 28-day mortality in COVID-19 patients requiring oxygen therapy [20].Currently, the World Health Organization suggested using Tocilizumab in severe or critical COVID-19 patients who exhibit signs of desaturation in room air, severe respiratory distress, ARDS, require life-sustaining treatment, sepsis and septic shock [21]. However, Tocilizumab may also exacerbate bacterial infections in COVID-19 patients, which could restrict its usage. In the RECOVERY trial, 16% of patients in the Tocilizumab group ultimately did not receive this treatment [20]. Tocilizumab would be administered only to COVID-19 patients with higher severity and fewer signs of complicating bacterial infection. This observation may reflect a higher severity of illness among patients in the antifibrotic group.

Nintedanib functions by binding to intracellular ATP pockets and inhibiting profibrotic signaling pathways, including platelet-derived growth factor, fibroblast growth factor, vascular endothelial growth factor, and transforming growth factor-beta (TGF-β) [10]. Similarly, pirfenidone regulates pro-fibrotic and pro-inflammatory cytokines such as TGF-β, tumor necrosis factor-α, interferon γ, interleukin-1β, and interleukin-6, thus inhibiting fibroblast proliferation and collagen synthesis [22, 23]. Moreover, pirfenidone has demonstrated a capacity to reduce ACE receptor expression, which is considered a major cellular receptor for SARS-CoV-2 virus entry [24]. Notably, case reports have indicated successful treatment outcomes using nintedanib for COVID-19-related ARDS [25, 26]. While ongoing studies on the treatment of COVID-19 patients with acute respiratory failure or pulmonary fibrosis exist, only limited published data are currently available. Umemura et al. observed that nintedanib significantly shortened the duration of mechanical ventilation and reduced the high attenuation area percentage on computed tomography volumetry in COVID-19 patients admitted to the ICU and requiring mechanical ventilation. However, no significant differences in 28-day mortality were found [27]. Similarly, Zhang et al. demonstrated that pirfenidone significantly decreased inflammatory biomarkers, including interleukin-2R, tumor necrosis factor-alpha (TNF-α), and D-dimer, although clinical parameters such as clinical improvement time, duration of oxygen therapy, time from randomization to death, and interstitial changes on CT images exhibited insignificant improvement [28].

Epithelial injury followed by a subsequent fibroproliferative cascade has been recognized as a key pathogenic mechanism of pulmonary fibrosis shared between COVID-19-related ARDS and idiopathic pulmonary fibrosis [29–32]. Type 2 alveolar epithelial cells (ATII cells) plays a crucial role in IPF development [33, 34, 35, 36]. SARS-CoV-2 enters lower respiratory tract cells via the angiotensin-converting enzyme 2 (ACE2) receptor in conjunction with transmembrane protease serine 2, expressed by ATII cells. Inadequate responses of ATII cells to lung injury lead to aberrant tissue repair, characterized by fibroblast activation, collagen deposition, connective tissue accumulation, and angiogenesis [37, 38]. Both idiopathic pulmonary fibrosis and COVID-19-induced pulmonary fibrosis involve the renin-angiotensin system (RAS) in their disease progression. A pivotal player in this context, ACE2, interacts with other components of the RAS. ACE2-mediated SARS-CoV-2 entry into lung cells is believed to result in reduced ACE2 expression, disturbing the RAS system balance and subsequently triggering inflammation and fibrosis [39].

Dysregulation of microRNAs (miRNAs) has been implicated in the development of pulmonary fibrosis in COVID-19 patients, contributing to collagen deposition and myofibroblast transformation [40]. This miRNA imbalance has also been observed in idiopathic pulmonary fibrosis patients [41]. Lacedonia et al. analyzed the expression of exosomal miRNAs and confirmed the key involvement of a let-7d down-regulation and dysregulation of miR-16 in IPF [42]. Notably, Guiot et al. identified a total of 34 dysregulated miRNAs that overlapped between COVID-19 and idiopathic pulmonary fibrosis [40].

Despite the insightful findings, our study has certain limitations. The utilization of retrospective electronic records introduces inherent weaknesses. Lack of access to raw data prevents an accurate assessment of disease severity, identification of the causes of acute respiratory failure, determination of whether SARS-CoV-2 related pneumonia was presenting, specification of specific dosage and timing or the reasons for initiating antifibrotic treatment. Potential miscoding, inaccurate coding, or incomplete clinical information about comorbidities, socioeconomic status, and lifestyle habits may also introduce biases. Moreover, the TriNetX database lacks certain clinical information, such as ventilator-free days, ICU days, ventilator weaning rates, and hospitalization duration. Consequently, the efficacy of antifibrotic therapy cannot be adequately evaluated based on these parameters.

## Conclusions

This retrospective multi-institutional study highlights the potential benefits of novel antifibrotic agents, nintedanib and pirfenidone, in reducing mortality among COVID-19 patients with acute respiratory failure. This study offers important insights into the therapeutic potential of these agents in managing the complex pathogenesis of both COVID-19-induced pulmonary fibrosis and idiopathic pulmonary fibrosis. The findings underscore the significance of targeting fibroproliferative pathways and the RAS in mitigating inflammation and fibrosis. However, it is important to acknowledge the study’s limitations, particularly the retrospective nature of data analysis and the potential for biases. Future research and clinical trials are needed to further validate the efficacy of these antifibrotic agents and explore their precise mechanisms of action in the context of COVID-19 and acute respiratory failure.

### Electronic supplementary material

Below is the link to the electronic supplementary material.

Supplementary Material 1

Supplementary Material 2

Supplementary Material 3

Supplementary Material 4

Supplementary Material 5

Supplementary Material 6

Supplementary Material 7

Supplementary Material 8

Supplementary Material 9

Supplementary Material 10

Supplementary Material 11

Supplementary Material 12

Supplementary Material 13

Supplementary Material 14

## Data Availability

All data generated or analysed during this study are included in this published article and its supplementary information files.

## Abbreviations

COVID: 19-coronavirus disease 2019
ARDS: acute respiratory distress syndrome
ICU: intensive care unit
IPF: idiopathic pulmonary fibrosis
HCOs: healthcare organizations
HR: hazard ratio
CI: confidence interval
TGF: β-transforming growth factor beta
TNF: α-tumor necrosis factor-alpha
ATII cells: type 2 alveolar epithelial cells
RAS: renin-angiotensin system
ACE2: angiotensin-converting enzyme 2
miRNA: microRNA
