# Supplementary material for: The effect of antifibrotic agents on acute respiratory failure in COVID-19 patients: a retrospective cohort study from TriNetX US collaborative networks
Source: BMC Pulm Med. 2024 Apr 2;24:160. doi: 10.1186/s12890-024-02947-5 (PMC10986056; doi:10.1186/s12890-024-02947-5)
Supplement: Supplementary file 3 — Supplementary Material 3 [file 12890_2024_2947_MOESM3_ESM.docx]

## Additional file 3. [The](file:///G:\我的雲端硬碟\TriNetX研究計畫\COVID%20MV%20Antifibrotic\Manuscript\Revision%202\%20All%20data%20generated%20or%20analysed%20during%20this%20study%20are%20included%20in%20this%20published%20article%20and%20its%20supplementary%20information%20files.%20The) protocol, cohort definition, query criteria, statistical analysis plan and other relevant study materials comparing the pirfenidone treatment group and the control group.

# Introduction

TriNetX is the global federated health research network providing access to electronic medical records (diagnoses, procedures, medications, laboratory values, genomic information) across large healthcare organizations (HCOs). This report was run on the set of HCOs grouped into a network called US Collaborative Network. This network included 61 HCO(s).

This report describes a Compare Outcomes Analysis, generated by the TriNetX platform on Jan 30, 2024, 12:03:36 UTC. This analysis compared the outcomes of two cohorts: Cohort A (31 patients) named Pirfenidone treatment group and Cohort B (180,586 patients) named Control group.

# Methods

The analysis process includes two main steps: 1) Defining the cohorts through query criteria; 2) Setting up and running the analysis. Setting up the analysis requires definitions for the index event, outcomes criteria, and the time frame. Compare outcomes supports four analyses: Measures of Association, Survival, Number of Instances and Lab result distribution. These analyses have additional options that are listed in the Outcomes Definitions and Analyses Specifications section below.

## Cohorts definition

This section lists all terms used in the definitions of the two cohorts.

### Query Criteria for Pirfenidone treatment group:

This query was run on the network US Collaborative Network with 61 HCO(s) queried and 61 HCO(s) responded. A total of 17 provider(s) responded with patients. The final cohort included 31 patients who matched the query criteria listed in the table below.

|  | | | | | |
| --- | --- | --- | --- | --- | --- |
| Ungrouped terms | | | | | |
|  | must have |  | demographics | Age | Age (at least 20 years (most recent occurrence)) |
| Group 1 | | | | | |
|  | **Group 1A COVID-19** | | | | |
|  | must have | any of | diagnosis | UMLS:ICD10CM:U07.1 | COVID-19 |
|  |  |  | diagnosis | UMLS:ICD10CM:U07.2 | COVID-19, virus not identified (WHO) |
|  |  |  | diagnosis | UMLS:ICD10CM:J12.82 | Pneumonia due to coronavirus disease 2019 |
|  |  |  | laboratory | UMLS:LNC:94500-6 | SARS-CoV-2 (COVID-19) RNA [Presence] in Respiratory specimen by NAA with probe detection (labResult: Positive) |
|  |  |  | laboratory | UMLS:LNC:94309-2 | SARS-CoV-2 (COVID-19) RNA [Presence] in Specimen by NAA with probe detection (labResult: Positive) |
|  |  |  | laboratory | UMLS:LNC:94565-9 | SARS-CoV-2 (COVID-19) RNA [Presence] in Nasopharynx by NAA with non-probe detection (labResult: Positive) |
|  |  |  | laboratory | UMLS:LNC:94759-8 | SARS-CoV-2 (COVID-19) RNA [Presence] in Nasopharynx by NAA with probe detection (labResult: Positive) |
|  |  |  | laboratory | UMLS:LNC:95608-6 | SARS-CoV-2 (COVID-19) RNA [Presence] in Respiratory specimen by NAA with non-probe detection (labResult: Positive) |
|  |  |  | laboratory | UMLS:LNC:94845-5 | SARS-CoV-2 (COVID-19) RNA [Presence] in Saliva (oral fluid) by NAA with probe detection (labResult: Positive) |
|  |  |  | laboratory | UMLS:LNC:95406-5 | SARS-CoV-2 (COVID-19) RNA [Presence] in Nose by NAA with probe detection (labResult: Positive) |
|  | date constraint | | The terms in this group occurred on or before Aug 23, 2023 | | |
|  | event relationship | | Any instance of IPF occurred at least 1 day before the first instance of COVID-19 | | |
|  | **Group 1B IPF** | | | | |
|  | cannot have |  | diagnosis | UMLS:ICD10CM:J84.112 | Idiopathic pulmonary fibrosis |
| Group 2 | | | | | |
|  | **Group 2A COVID-19** | | | | |
|  | must have | any of | diagnosis | UMLS:ICD10CM:U07.1 | COVID-19 |
|  |  |  | diagnosis | UMLS:ICD10CM:U07.2 | COVID-19, virus not identified (WHO) |
|  |  |  | diagnosis | UMLS:ICD10CM:J12.82 | Pneumonia due to coronavirus disease 2019 |
|  |  |  | laboratory | UMLS:LNC:94500-6 | SARS-CoV-2 (COVID-19) RNA [Presence] in Respiratory specimen by NAA with probe detection (labResult: Positive) |
|  |  |  | laboratory | UMLS:LNC:94309-2 | SARS-CoV-2 (COVID-19) RNA [Presence] in Specimen by NAA with probe detection (labResult: Positive) |
|  |  |  | laboratory | UMLS:LNC:94565-9 | SARS-CoV-2 (COVID-19) RNA [Presence] in Nasopharynx by NAA with non-probe detection (labResult: Positive) |
|  |  |  | laboratory | UMLS:LNC:94759-8 | SARS-CoV-2 (COVID-19) RNA [Presence] in Nasopharynx by NAA with probe detection (labResult: Positive) |
|  |  |  | laboratory | UMLS:LNC:95608-6 | SARS-CoV-2 (COVID-19) RNA [Presence] in Respiratory specimen by NAA with non-probe detection (labResult: Positive) |
|  |  |  | laboratory | UMLS:LNC:94845-5 | SARS-CoV-2 (COVID-19) RNA [Presence] in Saliva (oral fluid) by NAA with probe detection (labResult: Positive) |
|  |  |  | laboratory | UMLS:LNC:95406-5 | SARS-CoV-2 (COVID-19) RNA [Presence] in Nose by NAA with probe detection (labResult: Positive) |
|  | date constraint | | The terms in this group occurred at any time | | |
|  | event relationship | | The first instance of MV occurred within 3 days before or up to 1 month after any instance of COVID-19 | | |
|  | **Group 2B ARF** | | | | |
|  | must have | any of | diagnosis | UMLS:ICD10CM:J96.00 | Acute respiratory failure, unspecified whether with hypoxia or hypercapnia |
|  |  |  | diagnosis | UMLS:ICD10CM:J96.0 | Acute respiratory failure |
|  |  |  | diagnosis | UMLS:ICD10CM:J96.01 | Acute respiratory failure with hypoxia |
|  |  |  | diagnosis | UMLS:ICD10CM:J96.02 | Acute respiratory failure with hypercapnia |
| Group 3 | | | | | |
|  | **Group 3A ARF** | | | | |
|  | must have | any of | diagnosis | UMLS:ICD10CM:J96.00 | Acute respiratory failure, unspecified whether with hypoxia or hypercapnia |
|  |  |  | diagnosis | UMLS:ICD10CM:J96.0 | Acute respiratory failure |
|  |  |  | diagnosis | UMLS:ICD10CM:J96.01 | Acute respiratory failure with hypoxia |
|  |  |  | diagnosis | UMLS:ICD10CM:J96.02 | Acute respiratory failure with hypercapnia |
|  | date constraint | | The terms in this group occurred at any time | | |
|  | event relationship | | Any instance of antifibrotic occurred on or after any instance of MV | | |
|  | **Group 3B antifibrotic** | | | | |
|  | must have |  | medication | NLM:RXNORM:1592254 | pirfenidone |
|  | cannot have |  | medication | NLM:RXNORM:1592737 | nintedanib |

### Query Criteria for Control group:

This query was run on the network US Collaborative Network with 61 HCO(s) queried and 61 HCO(s) responded. A total of 59 provider(s) responded with patients. The final cohort included 180,586 patients who matched the query criteria listed in the table below.

| Ungrouped terms | | | | | |
| --- | --- | --- | --- | --- | --- |
|  | must have |  | demographics | Age | Age (at least 20 years (most recent occurrence)) |
| Group 1 | | | | | |
|  | **Group 1A COVID-19** | | | | |
|  | must have | any of | diagnosis | UMLS:ICD10CM:U07.1 | COVID-19 |
|  |  |  | diagnosis | UMLS:ICD10CM:U07.2 | COVID-19, virus not identified (WHO) |
|  |  |  | diagnosis | UMLS:ICD10CM:J12.82 | Pneumonia due to coronavirus disease 2019 |
|  |  |  | laboratory | UMLS:LNC:94500-6 | SARS-CoV-2 (COVID-19) RNA [Presence] in Respiratory specimen by NAA with probe detection (labResult: Positive) |
|  |  |  | laboratory | UMLS:LNC:94309-2 | SARS-CoV-2 (COVID-19) RNA [Presence] in Specimen by NAA with probe detection (labResult: Positive) |
|  |  |  | laboratory | UMLS:LNC:94565-9 | SARS-CoV-2 (COVID-19) RNA [Presence] in Nasopharynx by NAA with non-probe detection (labResult: Positive) |
|  |  |  | laboratory | UMLS:LNC:94759-8 | SARS-CoV-2 (COVID-19) RNA [Presence] in Nasopharynx by NAA with probe detection (labResult: Positive) |
|  |  |  | laboratory | UMLS:LNC:95608-6 | SARS-CoV-2 (COVID-19) RNA [Presence] in Respiratory specimen by NAA with non-probe detection (labResult: Positive) |
|  |  |  | laboratory | UMLS:LNC:94845-5 | SARS-CoV-2 (COVID-19) RNA [Presence] in Saliva (oral fluid) by NAA with probe detection (labResult: Positive) |
|  |  |  | laboratory | UMLS:LNC:95406-5 | SARS-CoV-2 (COVID-19) RNA [Presence] in Nose by NAA with probe detection (labResult: Positive) |
|  | date constraint | | The terms in this group occurred between Dec 1, 2019 and Aug 23, 2023 | | |
|  | event relationship | | Any instance of IPF occurred at least 1 day before the first instance of COVID-19 | | |
|  | **Group 1B IPF** | | | | |
|  | cannot have |  | diagnosis | UMLS:ICD10CM:J84.112 | Idiopathic pulmonary fibrosis |
|  |  | or | medication | NLM:RXNORM:1592737 | nintedanib |
|  |  | or | medication | NLM:RXNORM:1592254 | pirfenidone |
| Group 2 | | | | | |
|  | **Group 2A COVID-19** | | | | |
|  | must have | any of | diagnosis | UMLS:ICD10CM:U07.1 | COVID-19 |
|  |  |  | diagnosis | UMLS:ICD10CM:U07.2 | COVID-19, virus not identified (WHO) |
|  |  |  | diagnosis | UMLS:ICD10CM:J12.82 | Pneumonia due to coronavirus disease 2019 |
|  |  |  | laboratory | UMLS:LNC:94500-6 | SARS-CoV-2 (COVID-19) RNA [Presence] in Respiratory specimen by NAA with probe detection (labResult: Positive) |
|  |  |  | laboratory | UMLS:LNC:94309-2 | SARS-CoV-2 (COVID-19) RNA [Presence] in Specimen by NAA with probe detection (labResult: Positive) |
|  |  |  | laboratory | UMLS:LNC:94565-9 | SARS-CoV-2 (COVID-19) RNA [Presence] in Nasopharynx by NAA with non-probe detection (labResult: Positive) |
|  |  |  | laboratory | UMLS:LNC:94759-8 | SARS-CoV-2 (COVID-19) RNA [Presence] in Nasopharynx by NAA with probe detection (labResult: Positive) |
|  |  |  | laboratory | UMLS:LNC:95608-6 | SARS-CoV-2 (COVID-19) RNA [Presence] in Respiratory specimen by NAA with non-probe detection (labResult: Positive) |
|  |  |  | laboratory | UMLS:LNC:94845-5 | SARS-CoV-2 (COVID-19) RNA [Presence] in Saliva (oral fluid) by NAA with probe detection (labResult: Positive) |
|  |  |  | laboratory | UMLS:LNC:95406-5 | SARS-CoV-2 (COVID-19) RNA [Presence] in Nose by NAA with probe detection (labResult: Positive) |
|  | date constraint | | The terms in this group occurred at any time | | |
|  | event relationship | | The first instance of MV occurred within 3 days before or up to 1 month after any instance of COVID-19 | | |
|  | **Group 2B ARF** | | | | |
|  | must have | any of | diagnosis | UMLS:ICD10CM:J96.00 | Acute respiratory failure, unspecified whether with hypoxia or hypercapnia |
|  |  |  | diagnosis | UMLS:ICD10CM:J96.0 | Acute respiratory failure |
|  |  |  | diagnosis | UMLS:ICD10CM:J96.01 | Acute respiratory failure with hypoxia |
|  |  |  | diagnosis | UMLS:ICD10CM:J96.02 | Acute respiratory failure with hypercapnia |
| Group 3 | | | | | |
|  | **Group 3A ARF** | | | | |
|  | must have | any of | diagnosis | UMLS:ICD10CM:J96.00 | Acute respiratory failure, unspecified whether with hypoxia or hypercapnia |
|  |  |  | diagnosis | UMLS:ICD10CM:J96.0 | Acute respiratory failure |
|  |  |  | diagnosis | UMLS:ICD10CM:J96.01 | Acute respiratory failure with hypoxia |
|  |  |  | diagnosis | UMLS:ICD10CM:J96.02 | Acute respiratory failure with hypercapnia |
|  | date constraint | | The terms in this group occurred at any time | | |
|  | event relationship | | The first instance of No antifibrotic occurred on or after any instance of MV | | |
|  | **Group 3B No antifibrotic** | | | | |
|  | cannot have |  | medication | NLM:RXNORM:1592737 | nintedanib |
|  |  | or | medication | NLM:RXNORM:1592254 | pirfenidone |

## Analysis Setup

This section contains the Index Event and Time Window definitions and a list of selected outcomes and the analyses.

### Index Event & Time Window Definitions

The index event defines the point in time when each patient in the cohort enters the analysis. To define an index event for the cohort, one or more criteria for the cohort must be selected. The index date for each patient within a cohort is the day on which the patient first met the selected criteria for the cohort (listed in the table below).

As the index event defines the earliest time point after which outcomes are analyzed, the time window defines the duration during which outcomes are analyzed. The time window can start on the same day as the index event or at any specified time interval after the index event. The time window can end any time after the start date. Outcomes are defined as diagnoses, medications, procedures, or laboratory values that happened in the time window starting after the first occurrence of the index event.

### Time Window Used in this Analysis

This analysis included outcomes that occurred in the time window that started on the same day as the first occurrence of the index event and ended 365 days after the first occurrence of the index event

The index event only includes events that occurred up to 20 years ago. Patients whose index event occurred 20 years or more ago are excluded. In this analysis, 0 patients in Cohort 1 and 0 patients in Cohort 2 were excluded because they met the index event more than 20 years ago.

### Index Events Used in this Analysis

Index events for the Compare Outcomes analysis were derived from the cohort definitions. Index events were defined separately for each cohort and were based on the criteria used in the original cohort definition.

The index event for Pirfenidone treatment group was defined as the following:

|  | | | | | |
| --- | --- | --- | --- | --- | --- |
| Group 1 | | | | | |
|  | **Group 1A COVID-19** | | | | |
|  | must have | any of | diagnosis | UMLS:ICD10CM:U07.1 | COVID-19 |
|  |  |  | diagnosis | UMLS:ICD10CM:U07.2 | COVID-19, virus not identified (WHO) |
|  |  |  | diagnosis | UMLS:ICD10CM:J12.82 | Pneumonia due to coronavirus disease 2019 |
|  |  |  | laboratory | UMLS:LNC:94500-6 | SARS-CoV-2 (COVID-19) RNA [Presence] in Respiratory specimen by NAA with probe detection (labResult: Positive) |
|  |  |  | laboratory | UMLS:LNC:94309-2 | SARS-CoV-2 (COVID-19) RNA [Presence] in Specimen by NAA with probe detection (labResult: Positive) |
|  |  |  | laboratory | UMLS:LNC:94565-9 | SARS-CoV-2 (COVID-19) RNA [Presence] in Nasopharynx by NAA with non-probe detection (labResult: Positive) |
|  |  |  | laboratory | UMLS:LNC:94759-8 | SARS-CoV-2 (COVID-19) RNA [Presence] in Nasopharynx by NAA with probe detection (labResult: Positive) |
|  |  |  | laboratory | UMLS:LNC:95608-6 | SARS-CoV-2 (COVID-19) RNA [Presence] in Respiratory specimen by NAA with non-probe detection (labResult: Positive) |
|  |  |  | laboratory | UMLS:LNC:94845-5 | SARS-CoV-2 (COVID-19) RNA [Presence] in Saliva (oral fluid) by NAA with probe detection (labResult: Positive) |
|  |  |  | laboratory | UMLS:LNC:95406-5 | SARS-CoV-2 (COVID-19) RNA [Presence] in Nose by NAA with probe detection (labResult: Positive) |
|  | date constraint | | The terms in this group occurred on or before Aug 23, 2023 | | |
|  | event relationship | | Any instance of IPF occurred at least 1 day before the first instance of COVID-19 | | |
|  | **Group 1B IPF** | | | | |
|  | cannot have |  | diagnosis | UMLS:ICD10CM:J84.112 | Idiopathic pulmonary fibrosis |
| Group 2 | | | | | |
|  | **Group 2A COVID-19** | | | | |
|  | must have | any of | diagnosis | UMLS:ICD10CM:U07.1 | COVID-19 |
|  |  |  | diagnosis | UMLS:ICD10CM:U07.2 | COVID-19, virus not identified (WHO) |
|  |  |  | diagnosis | UMLS:ICD10CM:J12.82 | Pneumonia due to coronavirus disease 2019 |
|  |  |  | laboratory | UMLS:LNC:94500-6 | SARS-CoV-2 (COVID-19) RNA [Presence] in Respiratory specimen by NAA with probe detection (labResult: Positive) |
|  |  |  | laboratory | UMLS:LNC:94309-2 | SARS-CoV-2 (COVID-19) RNA [Presence] in Specimen by NAA with probe detection (labResult: Positive) |
|  |  |  | laboratory | UMLS:LNC:94565-9 | SARS-CoV-2 (COVID-19) RNA [Presence] in Nasopharynx by NAA with non-probe detection (labResult: Positive) |
|  |  |  | laboratory | UMLS:LNC:94759-8 | SARS-CoV-2 (COVID-19) RNA [Presence] in Nasopharynx by NAA with probe detection (labResult: Positive) |
|  |  |  | laboratory | UMLS:LNC:95608-6 | SARS-CoV-2 (COVID-19) RNA [Presence] in Respiratory specimen by NAA with non-probe detection (labResult: Positive) |
|  |  |  | laboratory | UMLS:LNC:94845-5 | SARS-CoV-2 (COVID-19) RNA [Presence] in Saliva (oral fluid) by NAA with probe detection (labResult: Positive) |
|  |  |  | laboratory | UMLS:LNC:95406-5 | SARS-CoV-2 (COVID-19) RNA [Presence] in Nose by NAA with probe detection (labResult: Positive) |
|  | date constraint | | The terms in this group occurred at any time | | |
|  | event relationship | | The first instance of MV occurred within 3 days before or up to 1 month after any instance of COVID-19 | | |
|  | **Group 2B ARF** | | | | |
|  | must have | any of | diagnosis | UMLS:ICD10CM:J96.00 | Acute respiratory failure, unspecified whether with hypoxia or hypercapnia |
|  |  |  | diagnosis | UMLS:ICD10CM:J96.0 | Acute respiratory failure |
|  |  |  | diagnosis | UMLS:ICD10CM:J96.01 | Acute respiratory failure with hypoxia |
|  |  |  | diagnosis | UMLS:ICD10CM:J96.02 | Acute respiratory failure with hypercapnia |

The index event for Control group was defined as the following:

|  | | | | | |
| --- | --- | --- | --- | --- | --- |
| Group 1 | | | | | |
|  | **Group 1A COVID-19** | | | | |
|  | must have | any of | diagnosis | UMLS:ICD10CM:U07.1 | COVID-19 |
|  |  |  | diagnosis | UMLS:ICD10CM:U07.2 | COVID-19, virus not identified (WHO) |
|  |  |  | diagnosis | UMLS:ICD10CM:J12.82 | Pneumonia due to coronavirus disease 2019 |
|  |  |  | laboratory | UMLS:LNC:94500-6 | SARS-CoV-2 (COVID-19) RNA [Presence] in Respiratory specimen by NAA with probe detection (labResult: Positive) |
|  |  |  | laboratory | UMLS:LNC:94309-2 | SARS-CoV-2 (COVID-19) RNA [Presence] in Specimen by NAA with probe detection (labResult: Positive) |
|  |  |  | laboratory | UMLS:LNC:94565-9 | SARS-CoV-2 (COVID-19) RNA [Presence] in Nasopharynx by NAA with non-probe detection (labResult: Positive) |
|  |  |  | laboratory | UMLS:LNC:94759-8 | SARS-CoV-2 (COVID-19) RNA [Presence] in Nasopharynx by NAA with probe detection (labResult: Positive) |
|  |  |  | laboratory | UMLS:LNC:95608-6 | SARS-CoV-2 (COVID-19) RNA [Presence] in Respiratory specimen by NAA with non-probe detection (labResult: Positive) |
|  |  |  | laboratory | UMLS:LNC:94845-5 | SARS-CoV-2 (COVID-19) RNA [Presence] in Saliva (oral fluid) by NAA with probe detection (labResult: Positive) |
|  |  |  | laboratory | UMLS:LNC:95406-5 | SARS-CoV-2 (COVID-19) RNA [Presence] in Nose by NAA with probe detection (labResult: Positive) |
|  | date constraint | | The terms in this group occurred between Dec 1, 2019 and Aug 23, 2023 | | |
|  | event relationship | | Any instance of IPF occurred at least 1 day before the first instance of COVID-19 | | |
|  | **Group 1B IPF** | | | | |
|  | cannot have |  | diagnosis | UMLS:ICD10CM:J84.112 | Idiopathic pulmonary fibrosis |
|  |  | or | medication | NLM:RXNORM:1592737 | nintedanib |
|  |  | or | medication | NLM:RXNORM:1592254 | pirfenidone |
| Group 2 | | | | | |
|  | **Group 2A COVID-19** | | | | |
|  | must have | any of | diagnosis | UMLS:ICD10CM:U07.1 | COVID-19 |
|  |  |  | diagnosis | UMLS:ICD10CM:U07.2 | COVID-19, virus not identified (WHO) |
|  |  |  | diagnosis | UMLS:ICD10CM:J12.82 | Pneumonia due to coronavirus disease 2019 |
|  |  |  | laboratory | UMLS:LNC:94500-6 | SARS-CoV-2 (COVID-19) RNA [Presence] in Respiratory specimen by NAA with probe detection (labResult: Positive) |
|  |  |  | laboratory | UMLS:LNC:94309-2 | SARS-CoV-2 (COVID-19) RNA [Presence] in Specimen by NAA with probe detection (labResult: Positive) |
|  |  |  | laboratory | UMLS:LNC:94565-9 | SARS-CoV-2 (COVID-19) RNA [Presence] in Nasopharynx by NAA with non-probe detection (labResult: Positive) |
|  |  |  | laboratory | UMLS:LNC:94759-8 | SARS-CoV-2 (COVID-19) RNA [Presence] in Nasopharynx by NAA with probe detection (labResult: Positive) |
|  |  |  | laboratory | UMLS:LNC:95608-6 | SARS-CoV-2 (COVID-19) RNA [Presence] in Respiratory specimen by NAA with non-probe detection (labResult: Positive) |
|  |  |  | laboratory | UMLS:LNC:94845-5 | SARS-CoV-2 (COVID-19) RNA [Presence] in Saliva (oral fluid) by NAA with probe detection (labResult: Positive) |
|  |  |  | laboratory | UMLS:LNC:95406-5 | SARS-CoV-2 (COVID-19) RNA [Presence] in Nose by NAA with probe detection (labResult: Positive) |
|  | date constraint | | The terms in this group occurred at any time | | |
|  | event relationship | | The first instance of MV occurred within 3 days before or up to 1 month after any instance of COVID-19 | | |
|  | **Group 2B ARF** | | | | |
|  | must have | any of | diagnosis | UMLS:ICD10CM:J96.00 | Acute respiratory failure, unspecified whether with hypoxia or hypercapnia |
|  |  |  | diagnosis | UMLS:ICD10CM:J96.0 | Acute respiratory failure |
|  |  |  | diagnosis | UMLS:ICD10CM:J96.01 | Acute respiratory failure with hypoxia |
|  |  |  | diagnosis | UMLS:ICD10CM:J96.02 | Acute respiratory failure with hypercapnia |

### Survival Analysis

The Kaplan-Meier Analysis estimates probability of the outcome at a respective time interval (daily time interval is used in this analysis). In order to account for the patients who exited the cohort during the analysis period, and therefore should not be included in the analysis, censoring is applied. In this analysis, patients are removed from the analysis (censored) after the last fact in their record.

The output summary includes: Patients in each Cohort (count of patients meeting query criteria); Patients with Outcome (of the patients in the cohort, count of patients that had the outcome in the time window); Median Survival (the number of days when the survival drops below 50%; the “-” indicates that survival does not drop below 50% during the time window); and Survival Probability at End of Time Window (the % survival at the end of the time window). In addition, Log-Rank test, Hazard Ratio and test for Proportionality.

### Outcome Definitions

Table below outlines the definitions for each outcome and the analysis specifications. For outcome definitions consisting of more than one term, at least one term must match.

| Unnamed Outcome | | | | |
| --- | --- | --- | --- | --- |
|  | **Outcome definition** | | | |
|  | | Demographics | Deceased | Deceased |
|  | **Settings for the performed analyses** | | | |
|  | | Risk analysis | | excluding patients with outcome prior to the time window |
|  | | Kaplan - Meier survival analysis | | excluding patients with outcome prior to the time window |
